# Protective role of p120‐catenin on mitochondria by inhibiting NLRP3 in ventilator‐induced lung injury

**DOI:** 10.1111/jcmm.14595

**Published:** 2019-09-10

**Authors:** Ge Liu, Changping Gu, Mengjie Liu, Huan Liu, Dong Wang, Xiaobin Liu, Yuelan Wang

**Affiliations:** ^1^ Department of Anesthesiology Shandong Provincial Qianfoshan Hospital, Shandong University Jinan China

**Keywords:** mitochondria, NLRP3, p120‐catenin, pulmonary oedema, ventilator‐induced lung injury

## Abstract

Mitochondria supply energy to maintain the integrity of cell junctions. NLRP3, as the core component of the inflammatory response, is crucial in mechanical stretching. Mechanical stretching could activate NLRP3 and induce mitochondrial dysfunction. The relationship between p120 and mitochondria in ventilator‐induced lung injury (VILI) has not been elucidated. MLE‐12 cells and wild‐type male C57BL/6 mice were pre‐treated with MCC950 (specific and highly efficient inhibitor of NLRP3) or a p120 siRNA‐liposome complex. Then, the cells were subjected to 20% cyclic stretching, and the mice were subjected to mechanical ventilation at a high tidal volume. Cell lysates and lung tissues were obtained to detect the expression of NLRP3, p120, TLR4 pathway components, IL‐6 and IL‐1β, to determine the functions and structures of mitochondria, and the wet/dry ratio of the lung, and to perform pathological staining and an Evans blue dye assay. Mechanical stretching could increase the levels of NLRP3, ROS and damaged mitochondria, while these changes could be reversed by MCC950. Moreover, p120 prevented the activation of NLRP3 and regulated NLRP3 by inhibiting the TLR4 pathway and ROS production. Additionally, p120 played a vital role in protecting mitochondrial structures and functions after mechanical stretching. Taken together, these findings suggest that p120 depletion during mechanical stretching aggravates mitochondrial dysfunction by activating NLRP3, which indicates that p120 has a protective role on mitochondria in VILI by inhibiting NLRP3 activation.

## 1 INTRODUCTION

Mechanical ventilation has become an essential therapy to save lives in clinical practice. However, improper ventilation parameters and modes can easily cause damage to the airway and pulmonary alveoli, which is known as ventilator‐induced lung injury (VILI).^1^, ^2^

Pulmonary oedema is the common manifestation of lung injury induced by various mechanisms. The formation of pulmonary oedema can affect the exchange of gases in the blood, lead to hypoxia of the body and, even finally, induce multiple organ dysfunction syndrome (MODS).^3^, ^4^, ^5^ Thus, the prevention of pulmonary oedema is the key to avoiding the occurrence of VILI, and the early prevention of pulmonary oedema can inhibit or prevent the subsequent series of reactions and damage, which means that preventing pulmonary oedema is the key link to protect against VILI.

Previous studies have shown that the complete structure of pulmonary epithelial cells, pulmonary vascular endothelial cells and normal cell junctions is responsible for the permeability of alveolar membranes.^6^, ^7^ One of the primary causes of pulmonary oedema is the increased lung permeability induced by the destruction of the alveolar membrane, while maintaining the integrity and function of pulmonary epithelial cells or pulmonary vascular endothelial cells requires an appropriate amount of energy.^8^, ^9^ Mitochondria provide energy for the activities of cells, and their involvement in processes such as lipid biosynthesis and trafficking, calcium homeostasis, reactive oxygen species (ROS) production and autophagy has been experimentally confirmed.^10^, ^11^ Therefore, mitochondria play important roles in maintaining the homeostasis of the cells. However, whether mitochondria are damaged during VILI, the mechanism of this process and how to prevent this damage still need to be further explored.

Previous studies have reported that the NLRP3 inflammasome is involved in inflammation and VILI and that NLRP3 is closely associated with mitochondria.^12^, ^13^, ^14^ In response to external changes, such as electricity, LPS or other stimuli, NLRP3 interacts with pro–caspase‐1 through ASC, which leads to the activation of caspase‐1. Activated caspase‐1 promotes the cleavage and maturation of pro‐inflammatory cytokines in the cytoplasm (pro–interleukin (IL)‐1β, pro–IL‐18 and IL‐33). Pro–IL‐1β is cleaved by caspase‐1, and then, mature IL‐1β is released.^15^, ^16^, ^17^, ^18^ Whether the activation of NLRP3 inflammasomes can directly affect mitochondrial structure and function during VILI is not clearly known.

Previous studies have revealed that p120 could block the TLR4 pathway and RhoA GTPases in the body's anti‐inflammatory response and p120 was also a key adherent junction protein in maintaining the integrity of the cell junction in VILI.^19^, ^20^ Moreover, p120 degradation activated RhoA, which indicated that p120 could prevent pulmonary damage during VILI.^5^, ^6^ However, it remains unknown whether mitochondrial structure and functions were affected in VILI and whether NLRP3 participated in that process. We still do not know whether there are some connections between p120 and mitochondria. Therefore, the relationship among p120, NLRP3 and mitochondria in VILI needs to be further explored. In this study, we used both in vivo and in vitro models of VILI to explore the protective role and mechanisms of p120 on mitochondria in VILI. We observed that p120 could down‐regulate NLRP3 activation by inhibiting the TLR4 signalling pathway and ROS production under oxidative stress injury, thus reducing the damage to mitochondrial structures and functions. All our findings collectively provide a novel insight into the role of p120 in the pathophysiology of VILI and suggest that mitochondria are potential therapeutic targets for preventing VILI.

## 2 MATERIALS AND METHODS

### 2.1 Reagents and kits

Cell culture medium (DMEM/F‐12) was purchased from HyClone, and foetal bovine serum (FBS) was purchased from Gibco. Rabbit TLR4 polyclonal antibody, rabbit p120 monoclonal antibody and rabbit ICAM1 monoclonal antibody were purchased from Abcam. Rabbit NLRP3 monoclonal antibody, rabbit β‐actin monoclonal antibody, rabbit NF‐κB monoclonal antibody and rabbit phospho–NF‐κB monoclonal antibody were purchased from CST. An effective and selective inhibitor of NLRP3, MCC950, was purchased from Selleck. Mouse alveolar epithelial cells (MLE‐12) were purchased from American Type Culture Collection. p120 siRNA was purchased from GenePharma. The transfection regent, Lipofectamine 2000, was purchased from Invitrogen. The mitochondrial extraction kit was from Invent. The mitochondrial membrane potential (MMP) detection kits and the ROS assay kits were both from Beyotime Biotechnology. The IL‐1β and IL‐6 enzyme‐linked immunosorbent assay (ELISA) kits were purchased from R&D.

### 2.2 Cell culture and treatments

MLE‐12 cells were plated at a density of 2.5 × 10^5 cells/mL on culture dishes or BioFlex plates coated with collagen protein I coated in DMEM/F‐12 with 10% FBS at 37°C and 5% CO_2_, and incubated for 24‐48 hours. MLE‐12 cell monolayers were serum‐deprived for 2 hours prior to cyclic stretching treatments.

For inhibitor studies, when the MLE‐12 cell monolayers on BioFlex plates had grown 85%‐95%, the cells were serum‐deprived for 2 hours prior to experiments and treated with 1 μmol/L MCC950, for 1 hours at 37°C and 5% CO_2_ for cyclic stretching testing.^21^, ^22^

For the transient transfection of p120 siRNA, siRNA was synthesized by GenePharma. Then, the siRNA and Lipofectamine 2000 (Invitrogen) were diluted with 200 μL basal medium, DMEM/F‐12 without FBS. p120 siRNA (50 nmol/L) and the transfection reagent Lipofectamine 2000 were each diluted with DMEM/F‐12 and mixed 5 minutes later. Then, the mixture was incubated at room temperature for 20 minutes so that it could be added to the cell culture medium before further incubation at 37°C and 5% CO_2_ for 48 hours. Transfection efficiency was measured by Western blot analysis.

Alveolar epithelial cell monolayers on flexible membranes were mounted for cyclic stretching using the FX‐5000T Flexcell Tension System (Flexcell International) equipped with a 25‐mm BioFlex loading station. Cyclic stretching was conducted with a 20% change in the basement surface area at a frequency of 30 cycles/minute (0.5 Hz) and a stretch‐to‐relaxation ratio of 1:1 applied in a cyclic manner. These surface area changes correspond to 80% of the total lung capacity.^5^ The flexible cell‐covered elastomer membranes were stretched by applying an oscillating vacuum to the underside of the membranes. The duration, amplitude and frequency of the applied stretches were controlled by a computer. Unstretched cells were used as the control group (C group). Comparisons were made between the C group and treatment groups.

### 2.3 Animals and treatments

Animal experiments were approved by the Animal Ethics Committee of Shandong University. Seventy‐two healthy wild‐type (WT) male C57BL/6 mice (25‐30 g) were obtained from the Beijing Vital River Laboratory Animal Technology. The mice were housed under specific pathogen‐free conditions and used in experiments at 8‐12 weeks of age.

For the inhibitor studies, the mice were randomly divided into the following four groups (n = 6 in each group): C group; high tidal volume group (MV group); DMSO group (D group); and high tidal volume +NLRP3 inhibitor (MCC950) group (MV + M group). Mechanical ventilation was not conducted in the C and D groups. The other two groups were mechanically ventilated for 4 hours using an ALC‐V8 animal ventilator. The tidal volume was 28 mL/kg in the MV and MV + M groups.

For another study, the mice were randomly divided into the following four groups (n = 6 in each group): WT group; MV group; p120 KO group; and high tidal volume + p120 KO group (p120 KO + MV group). Mechanical ventilation was not conducted in the WT and p120 KO groups. The other two groups were mechanically ventilated for 4 hours. The tidal volume was 28 mL/kg in the MV and p120 KO + MV groups.

To decrease the level of p120 in vivo, we injected liposome‐based ingredients in the retinal vein plexus for the systemic and targeted delivery of p120 siRNA. Each animal received 300 μL siRNA‐liposome complexes consisting of 150 μL p120 siRNA (7.5 nmol) dilution and 150 μL liposomes through injection in the retinal vein plexus, and a mouse required three repeated injections over 2 weeks. Then, mouse lungs were obtained to detect p120 KO efficiency by using Western blot analysis. Several days after injecting p120 siRNA, the mice appeared to have inflammatory bowel diseases, such as intestinal adhesion, which was similar to Karayiannakis's study.^23^

The mice in the ventilation groups were anaesthetized by an intraperitoneal injection of pentobarbital sodium (60 mg/kg) and ketamine (80 mg/kg). Anaesthesia was maintained by infusing pentobarbital at 15 mg/kg every 30 minutes via the tail vein. Muscle relaxation was maintained with pancuronium (2 mg/kg/h). The ventilation parameters were set as follows: respiratory rate of 68 times/minute; I/E ratio of 1:2; and inspired oxygen fraction of 21%. Mice in MV + M group were pre‐treated with MCC950 (4 mg/kg) 1 hours before anaesthesia.^18^, ^19^ After 4 hours of ventilation, the mice were killed by exsanguination of arterial blood. The lungs were removed, and the upper lobe of the right lung was quickly frozen in liquid nitrogen, which was used for Western blot analysis and MMP detection. The remnant right lung tissue was fixed in 4% paraformaldehyde for 48‐72 hours for haematoxylin and eosin (HE) staining. The left lung was used to calculate the pulmonary wet/dry (W/D) weight ratio to quantify the magnitude of pulmonary oedema. First, the weight of the wet lung was determined. Then, the tissues were incubated in a 70°C incubator for 72 hours to obtain the dry weight. The other mice were used to observe lung permeability by using Evans blue staining after experiments, and bronchial alveolar lavage fluid was collected to detect inflammatory factors.

### 2.4 Western blot analysis

Pulmonary tissue homogenate and MLE‐12 monolayer cultures were dissolved on ice with prepared RIPA buffer containing 1 mmol/L phosphatase inhibitor and 1 mmol/L PMSF for 30 minutes. The final lysate was collected, and the protein concentration was determined using a bicinchoninic acid protein assay kit (Beyotime). Equal amounts of protein were loaded for 10% SDS‐PAGE, and then, the proteins were transferred to polyvinylidene fluoride membranes. The membranes were blocked in 5% skim milk in TBS containing 0.1% Tween‐20 (pH 8.8) for 2 hours at room temperature. Then, the membranes were incubated with primary antibodies at 4°C overnight. The concentrations of all primary antibodies were as follows: anti‐p120, 1:5000 dilution (Abcam); anti‐phosphorylation NF‐κB and anti–NF‐κB, 1:1000 dilution (CST); anti‐NLRP3, 1:500 dilution (CST); and anti‐TLR4, 2 μg/mL dilution (Abcam). The membranes were washed with TBST and then incubated with appropriate horseradish peroxidase‐conjugated secondary antibody for 2 hours at room temperature. Finally, the protein bands were detected by chemiluminescence using an enhanced chemiluminescence (ECL) system. The density of all proteins was analysed using ImageJ software. Western blot bands were simply processed with Adobe Photoshop CS6.

### 2.5 Transmission electron microscopy observation

MLE‐12 monolayer cultures were gently scraped off the culture plates and quickly placed in a 3% glutaraldehyde solution, at 4℃ for 2 hours. After repeated rinsing with PBS, cell samples were fixed with 1% osmic acid under 4℃ for 2 hours. Then, the cell samples were dehydrated with different concentrations of ethanol and acetone. Afterwards, the samples were fully soaked and embedded in a fixation and embedding medium. Then, the samples were cut into ultrathin sections of 60 nm and stained by using the uranium acetate‐lead citrate double‐dyeing process to increase the contrast among cell structures. Finally, the structure of mitochondria was observed under a transmission electron microscope (TEM).

### 2.6 Immunofluorescence, haematoxylin and eosin and Evans blue staining

For the in vitro study, after cyclic stretching, the cells were washed with PBS three times and then fixed in 4% paraformaldehyde for 15 minutes before permeabilization with 0.5% Triton X‐100 for 20 minutes. The cells were incubated in 5% bovine serum albumin (Solarbio) for 1 hour. Then, the cells were incubated with rabbit anti‐NLRP3 (1:100) and goat anti–caspase‐1 polyclonal antibodies (1:100 dilution) overnight at 4°C. Subsequently, the cells were incubated with secondary antibodies including donkey anti‐rabbit IgG (H + L) (red, 1:500 dilution) (Abbkine) and donkey anti‐goat IgG (H + L) (green, 1:500 dilution) (Abbkine) for 1 hour at room temperature. Finally, 4', −6‐diamidino‐2‐phenylindole (DAPI, 2 μg/mL) was added for nuclear staining for 5 minutes.

For the in vivo study, lung tissues were obtained and then soaked in a fixation solution (10% formalin) for at least 48 hours. Fixed lung tissues were dehydrated and embedded in paraffin. Next, paraffin‐embedded tissue was sectioned at a thickness of 4 um and then dewaxed. The pathological changes in lung tissues were investigated with HE staining.

Evans blue (Beyotime, Shanghai, CHN) staining was used to indicate the permeability of pulmonary tissue. After treatment, Evans blue fluid（0.3 mL per mouse) was injected into the mouse retinal vein plexus. Three minutes later, the abdominal aorta was cut and bled. The chest was quickly opened, and the left auricle was cut off. One catheter was inserted into the right ventricle towards the pulmonary artery. Then, 10 mL cold normal saline was continuously injected into the catheter to remove the blood from the lung of the mouse with limited damage. An entire mouse lung was quickly isolated.

### 2.7 Wet/dry weight ratio

The W/D weight ratio of the lung was obtained to quantify pulmonary oedema. After mechanical ventilation, one lobe of the right lung tissue was obtained after removing the liquid from the surface of the mouse lung. Lung tissues were weighed and then dried in an oven (60°C for 72 hours). Finally, we measured the dry weight and calculated the W/D ratio.

### 2.8 Cytokine assays

The levels of IL‐1β and IL‐6 in bronchoalveolar lavage fluid (BALF) were detected using commercial ELISA kits (R&D Systems) according to the manufacturer's instructions. Each value represents the mean of triplicate determinations.

### 2.9 Mitochondrial membrane potential and reactive oxygen species detection with flow cytometry

JC‐1 is a kind of fluorescent probe wildly used to detect MMP. When the MMP increases, JC‐1 forms J‐aggregates, which produce red fluorescence. Inversely, JC‐1 exists in a monomeric form when the MMP is at a normal level. At the end of cyclic stretching, the cells were washed twice with PBS, and then, the cells in each well were incubated with 1 mL JC‐1 working solution for 20 minutes in a cell incubator at 37°C and 5% CO_2_. The JC‐1 working solution consisted of 50 µL JC‐1 (200×), 8 mL ultrapure water and 2 mL JC‐1 dye buffer (5×). Twenty minutes later, the supernatant in each well was removed and the cells were washed twice with JC‐1 dye buffer (1×). Then, the MLE‐12 cells were digested with pancreatic enzyme and resuspended in 0.5 mL JC‐1 dye buffer (1×). Finally, the fluorescent dye was detected by flow cytometry (FCM).

The fluorescent probe DCFH‐DA can freely pass through the cell membrane and is hydrolysed to DCFH by an esterase enzyme. Non‐fluorescent DCFH can be oxidized by intracellular ROS to fluorescent DCF, which is used to detect the level of ROS in the cells. DCFH‐DA was diluted with serum‐free medium to 1:1000. Cell culture medium was removed, and diluted DCFH‐DA (1 mL/well) was added. After 20 minutes of incubation in a cell incubator at 37°C and 5% CO_2_, the cells were washed with serum‐free medium for three times. Then, the MLE‐12 cells were digested with pancreatic enzyme and resuspended in 0.5 mL serum‐free medium in each well. Finally, the fluorescent dye was detected by FCM.

For MMP detection, 490 nm (excitation wavelength, EXW) and 530 nm (emission wavelength, EXW) were used to detect JC‐1 monomers. Additionally, 525 nm (EXW) and 590 nm (EMW) were used to detect JC‐1 aggregates. For ROS detection, 488 nm (EXW) and 525 nm (EMW) were used. FlowJo v10 was used to generate images.

### 2.10 Mitochondria extraction from lung tissue

After mechanical ventilation, a piece of fresh lung tissue (20‐30 mg) was mixed with 100 mg tissue dissociation beads, before it was placed in a filter cartridge. Buffer A (250 μL) was added to the filter, and the tissue was repeatedly pushed against the surface of the filter with twisting force and ground with a plastic rod for 1 minute. Then, the tube was incubated for 5 minutes.

The filter cartridge was capped and centrifuged at 16 000 × g for 20 seconds. Then, the filter was discarded and the pellet was resuspended by brief vortex. Next, the pellet was centrifuged at 3 000 rpm for 1 minute. The supernatant was carefully transferred to a fresh 2.0‐mL tube, and 400 μL buffer B was immediately added. The supernatant and buffer B were mixed and vortexed for 10 seconds. The mixture was centrifuged at 16 000 × g for 10 minutes. Subsequently, the supernatant was completely removed and the pellet was resuspended in 200 μL buffer B by vigorously vortexing for 10 seconds.

The tube was centrifuged at 10 000 rpm for 5 minutes, and then, the supernatant was transferred to a fresh 2.0‐mL tube, and 1.6 mL cold PBS was immediately added. The tube was centrifuged at 16 000 × g for 15 minutes. Finally, the supernatant was discarded and the isolated mitochondria were collected at the bottom of the tube.

### 2.11 Statistical analysis

Statistical analysis was performed with the SPSS 19.0 statistics package. One‐way analysis of variance (ANOVA) with a post hoc Turkey's pairwise comparison was used for statistical analysis among groups. Student's *t* test was performed for paired samples. All results are expressed as the mean ± SD *P* < .05 was considered statistically significant.

## 3 RESULTS

### 3.1 The NLRP3 inhibitor MCC950 rescued cyclic stretching‐induced mitochondrial damage

**Figure 1:**
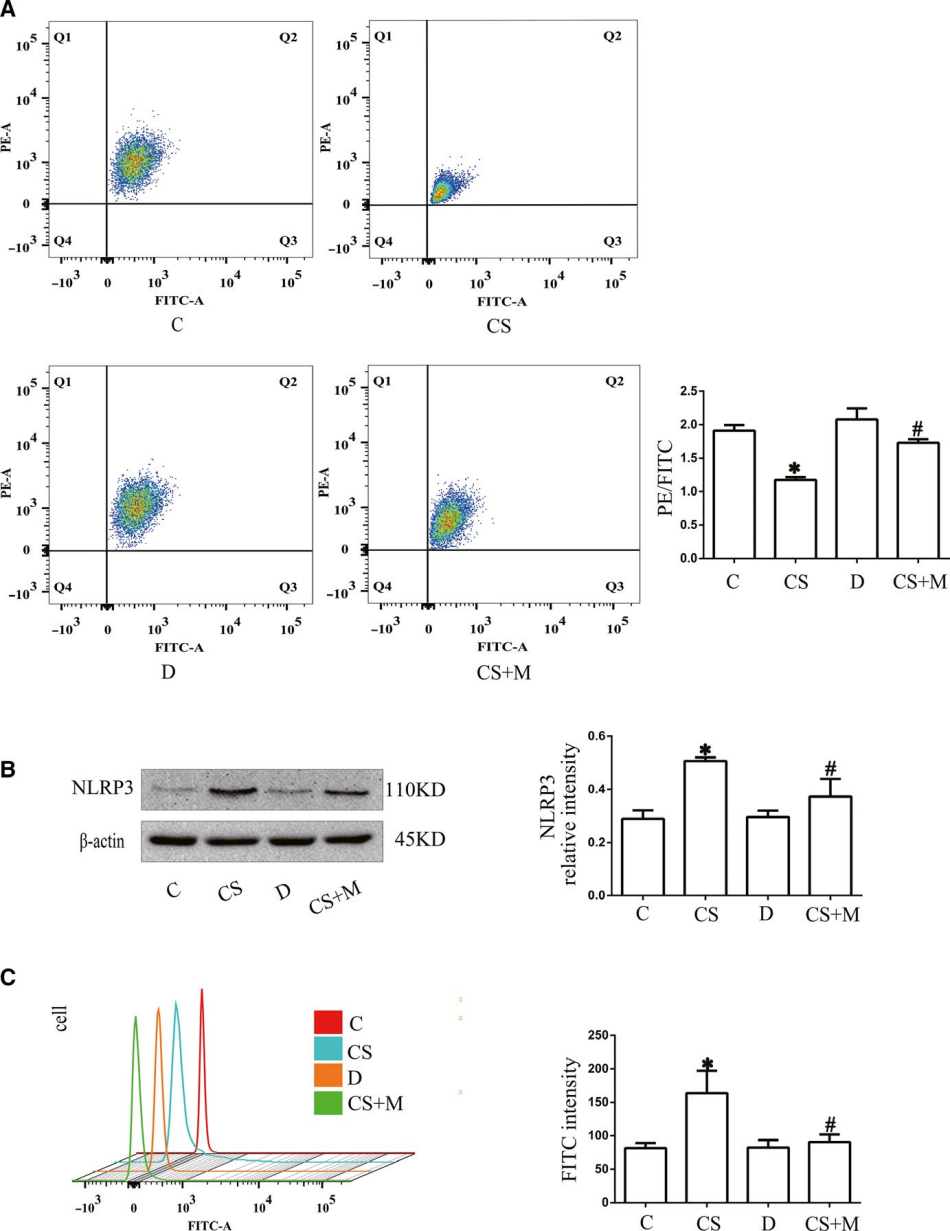
The effects of NLRP3 on mitochondria during cyclic stretching. MLE‐12 cells were pre‐treated with MCC950 (1 µmol/L) for 1 h before cyclic stretching. A, The level of MMP was detected by FCM. B, The expression of NLRP3 after cyclic stretching with or without MCC950 treatment was detected by Western blot analysis. C, The level of intracellular ROS production was detected by FCM. *P < .05 vs the C group, *#P* < .05 vs CS group. Experiments were repeated at least three times

To confirm the effects of NLRP3 on mitochondria during cyclic stretching, MLE‐12 cells were treated with the NLRP3 inhibitor MCC950. MLE‐12 cells were randomly divided into the following four groups: C group (with no treatment); cyclic stretching group (CS group, with 20% cyclic stretching for 4 hours); D group (treated with DMSO for 1 hours); and cyclic stretching + MCC950 group (CS + M group, pre‐treated with MCC950 for 1 hours before cyclic stretching for 4 hours). The cells in all groups were collected for the following experiments. NLRP3 was analysed by Western blot analysis, and MMP and intracellular ROS production were detected by FCM. The results suggested that NLRP3 expression, MMP and ROS production in the C group and D group were not significantly different (*P* > .05) (Figure 1A,B and 1). NLRP3 expression and ROS production were increased, and MMP was decreased in the CS group compared with the C group (*P* < .05) (Figure 1A,B and 1), and these results could be reversed by MCC950 treatment (*P* < .05) (Figure 1A,B and 1). MCC950 decreased NLRP3 expression and ROS production and increased MMP, while cyclic stretching had the opposite effect. This result indicated that cyclic stretching could induce mitochondrial damage and that NLRP3 played an important role in this process.

### 3.2 p120 prevented the activation of NLRP3 after cyclic stretching

**Figure 2:**
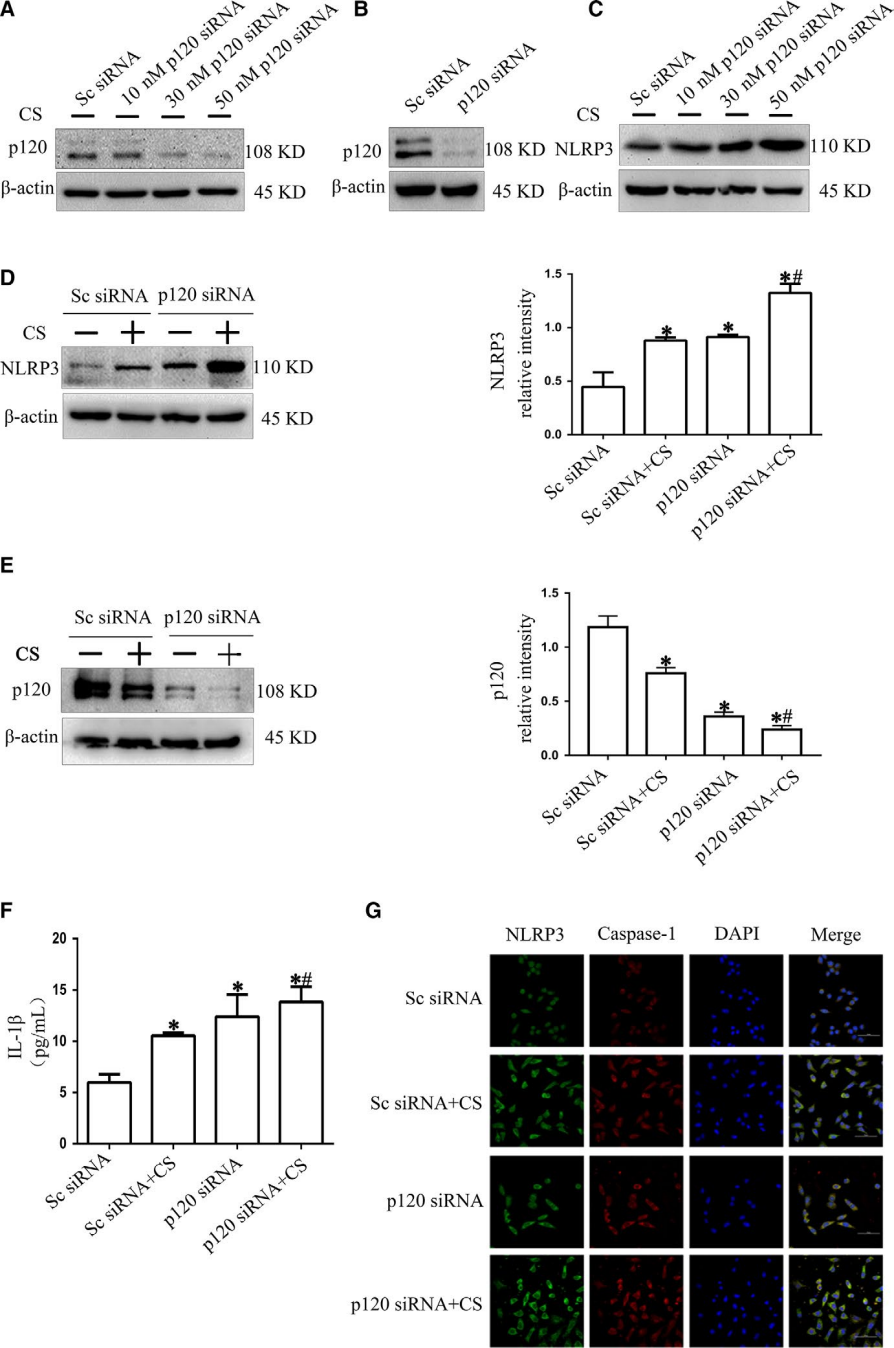
The relationship between p120 and NLRP3 during cyclic stretching. MLE‐12 cells were transfected with p120 siRNA. Forty‐eight hours post‐transfection, the cells were exposed to 20% cyclic stretching for 4 h. A, Successful transfection was detected by Western blot analysis. B, Optimum transfection concentration was verified by Western blot analysis. The p120 siRNA specifically knocked down p120 expression as demonstrated at the protein levels in a dose‐dependent manner. C, NLRP3 level was detected by Western blot. NLRP3 increased in a dose‐dependent manner after p120 depletion. D and E, The effect of p120 siRNA transfection on NLRP3. Loss of p120 could significantly increase the expression of NLRP3. F, IL‐1β was detected by ELISA. G, Immunofluorescence revealed the relationships among p120, NLRP3 and caspase‐1 after cyclic stretching in the p120 siRNA group. At the end of cyclic stretching, the cells were fixed, blocked and then incubated with NLRP3 and caspase‐1 primary antibody overnight at 4°C. Donkey anti‐rabbit IgG (red) and donkey anti‐goat IgG (green) were used as secondary antibodies. Nuclei were counterstained with DAPI (blue). Scale bars = 50 μm. *P < .05 vs the C group, *#P* < .05 vs the CS group. Experiments were repeated at least three times

To explore the role of p120 in the cyclic stretching‐induced dysfunction of the NLRP3 inflammasome, we depleted the p120 protein in MLE‐12 cells by p120 siRNA transfection. MLE‐12 cells were treated with 10 nmol/L, 30 nmol/L and 50 nmol/L p120 siRNA to choose the proper concentration for the following experiments (Figure 2A). Treatment with 50 nmol/L p120 siRNA was chosen as the best concentration, and the transfection efficiency was determined by Western blotting (Figure 2B). Western blot analysis revealed that NLRP3 levels increased in a dose‐dependent manner after p120 depletion (Figure 2C). NLRP3 expression was increased in the p120 siRNA group and CS group compared with the C group, and this effect was enhanced in the p120 siRNA group after 4 hours of 20% cyclic stretching (Figure 2D). Conversely, p120 expression was decreased in the p120 siRNA group compared with the C group, and this effect was enhanced in the p120 siRNA group after 4 hours of 20% cyclic stretching (Figure 2E). In addition, immunofluorescence suggested that the cells with p120 knocked down cells and 20% cyclic stretching showed a significant increase in both NLRP3 and caspase‐1 (Figure 2G). In addition to the activation of NLRP3, a product of the activated NLRP3 inflammasome, IL‐1β, increased as NLRP3 increased (Figure 2F). All of these results indicated that p120 played a crucial role in regulating NLRP3.

### 3.3 p120 regulated NLRP3 by inhibiting the TLR4 pathway and ROS activation after cyclic stretching

**Figure 3:**
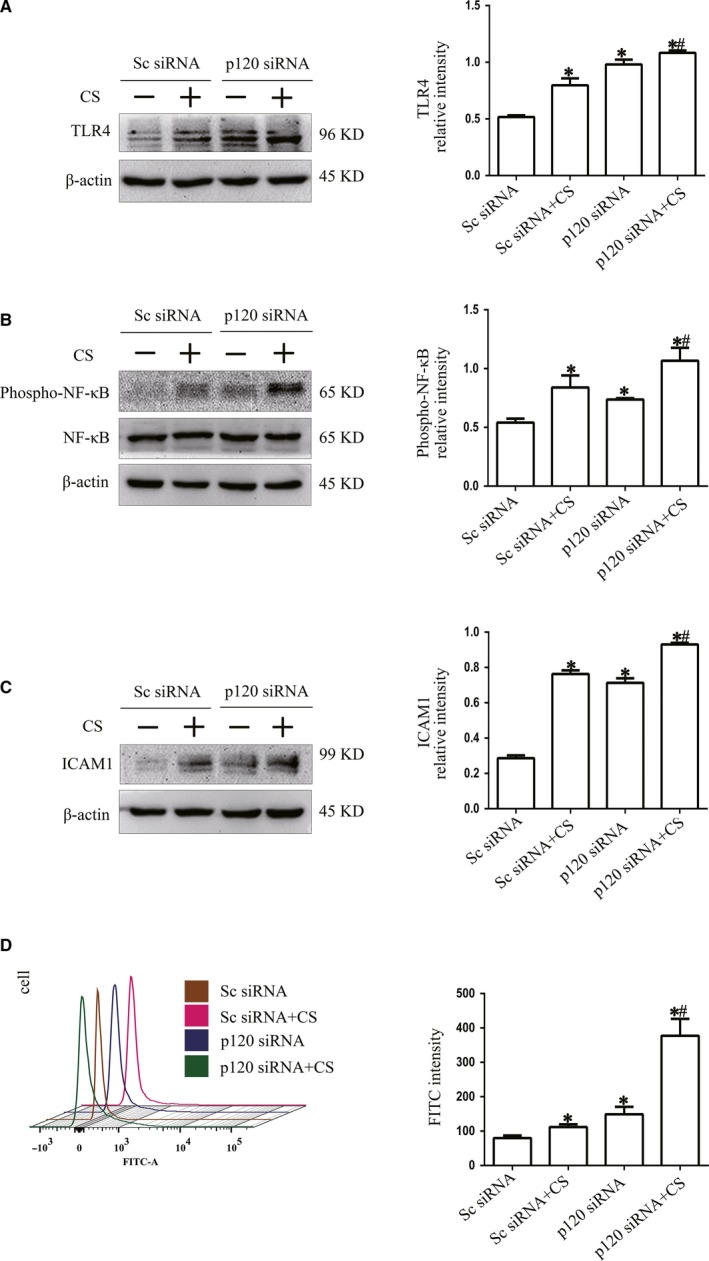
The regulatory role of p120 on the activation of NLRP3 and ROS. MLE‐12 cells were transfected with p120 siRNA before 20% cyclic stretching. A, B and C, Representative Western blot of TLR4 pathway proteins. The expression of TLR4, phospho–NF‐κB, NF‐κB and ICAM1 was detected by Western blot analysis. D, ROS activation was examined by FCM. *P < .05 vs the C group, *#P* < .05 vs the CS group. Experiments were repeated at least three times

To determine the regulatory mechanism of p120 in NLRP3 activation, we detected the expression of phospho–NF‐κB, NF‐κB and ICAM1. Western blot analysis revealed that the expression levels of TLR4, phospho–NF‐κB, NF‐κB and ICAM1 were all increased in the p120 siRNA group and CS group compared with the C group, while they were enhanced in the p120 siRNA group after 4 hours of 20% cyclic stretching (Figure 3A,B and 3). Similarly, FCM showed that ROS production was increased in the p120 siRNA group and CS group compared with the C group, and it was further enhanced in the p120 siRNA group after 4 hours of 20% cyclic stretching (Figure 3D). Therefore, the down‐regulation of p120, which was induced by cyclic stretching, could activate the TLR4 signalling pathway and oxidative stress injury, and this mechanism might be related to the cyclic stretching‐induced activation of NLRP3.

### 3.4 p120 played a vital role in protecting mitochondrial structures and functions after cyclic stretching

**Figure 4:**
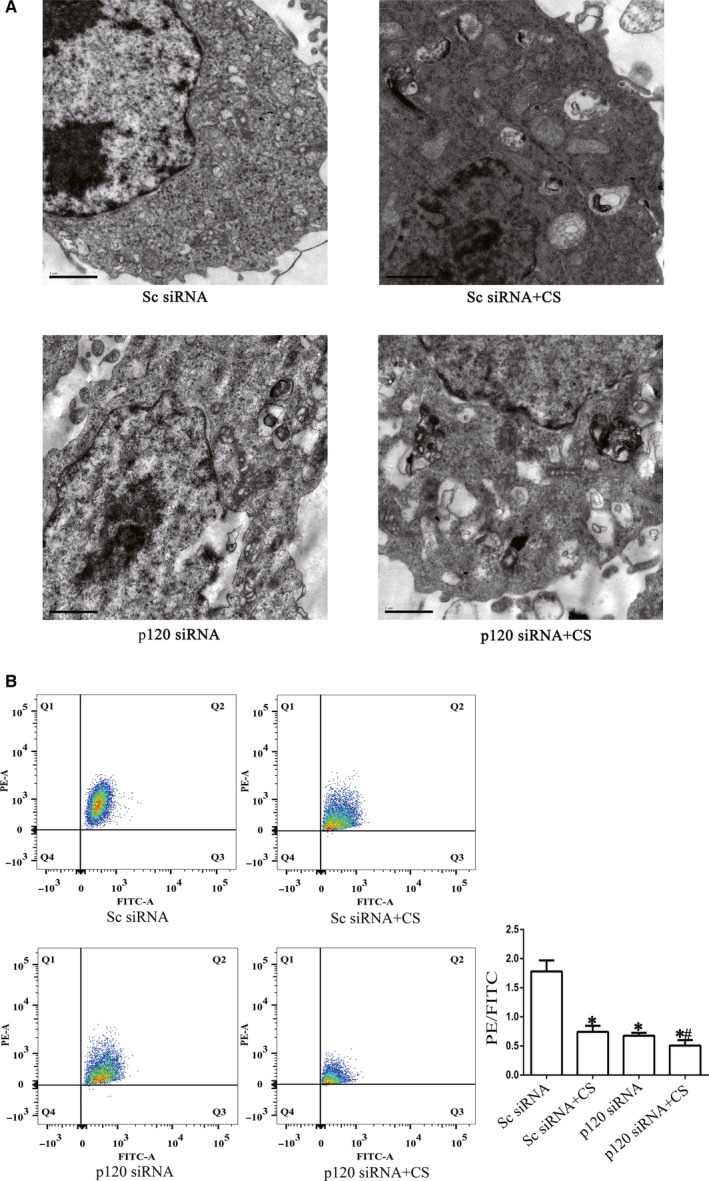
The effects of p120 on protecting mitochondrial structures and functions after cyclic stretching. MLE‐12 cells were transfected with p120 siRNA. Forty‐eight hours post‐transfection, the cells were exposed to 20% cyclic stretching for 4 h. A, Representative electron microscopy images of the four groups. Scale bars = 1 μm. B, MMP was examined by FCM. *P < .05 vs the C group, *#P* < .05 vs the CS group. Experiments were repeated at least three times

To determine the effect of p120 on mitochondria in VILI, we observed the subcellular structures of mitochondria under an electron microscope and detected the MMP by using FCM. Ultrastructural changes indicated mitochondrial damage and vacuole formation in the CS group and p120 siRNA group (Figure 4A). The mitochondria in the p120 siRNA + CS group showed more degenerative signs than those in the CS group, such as oedema of mitochondria, a decrease in mitochondrial crest and matrix density or an increase in vacuolation (Figure 4A).

MMP reflected early functional changes in mitochondria. We found that MMP was decreased in the CS group and p120 siRNA group compared with the C group, while the MMP of the p120 siRNA + CS group decreased more notably than that of the CS and p120 siRNA groups (Figure 4B).

### 3.5 p120 regulated mitochondria through NLRP3 in ventilator‐induced lung injury

Based on the cyclic stretching data, we further determined the mechanisms by which p120 regulates mitochondria and the protective role of p120 against VILI in vivo. Some mice were pre‐treated with MCC950 1 hours before mechanical ventilation, and the remaining mice were transfected with p120 siRNA or scrambled siRNA using p120 siRNA‐liposomes. Successful transfection was determined by Western blot analysis (Figure 6A a). After 4 hours of ventilation, BALF and lung tissues were obtained.

**Figure 5:**
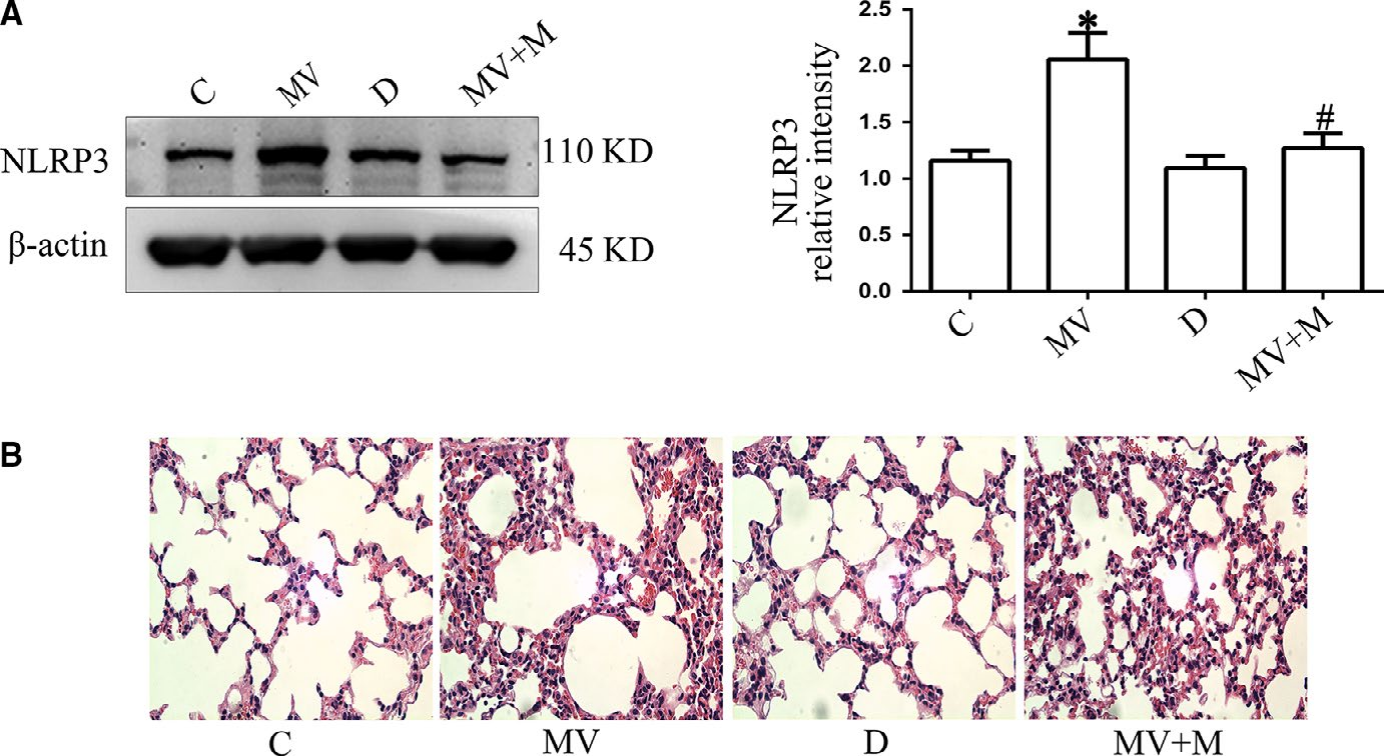
The role of NLRP3 in VILI in mice. Some mice were pre‐treated with MCC950 (specific inhibitor of NLRP3, 4 mg/kg) for 1 h before exposure to high tidal volume mechanical ventilation (MV) for 4 h. Mice without ventilation were regarded as the C group. A, The expression of NLRP3 was detected by Western blot analysis. NLRP3 was increased in the MV group compared with the C group, while this effect was reversed in the MCC950 pre‐treatment group. B, Representative HE staining images of the lung in the four groups: C group, MV group, D group and MV + M group (original magnification, ×400). *P < .05 vs the C group, *#P* < .05 vs the CS group. Experiments were repeated at least three times

**Figure 6:**
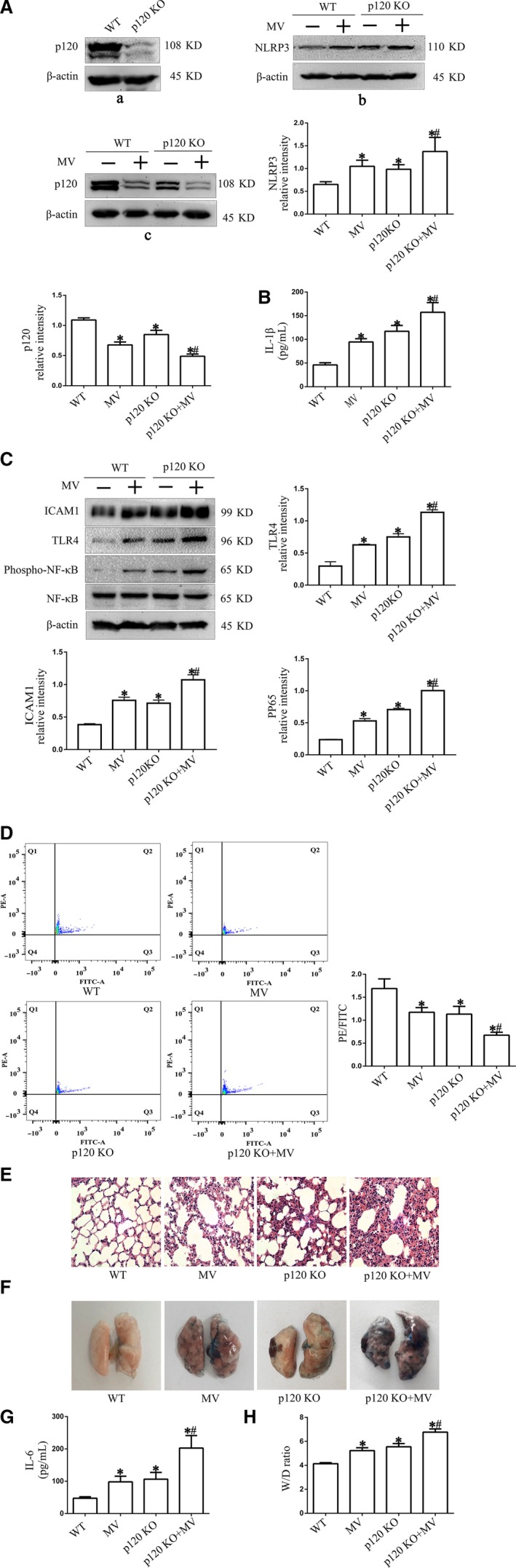
The role of p120 in VILI in mice. Some mice were injected with a complex composed of liposomes and p120 siRNA through the retinal vein plexus to knock down p120. Mice without ventilation were regarded as the C group. A, a shows the successful transfection of p120 siRNA as confirmed by Western blot analysis of lung homogenates from mice. b and c show the effect of p120 siRNA transfection on NLRP3. Loss of p120 could significantly increase the expression of NLRP3 in lung tissue. B, IL‐1β level in BALF. C, Representative Western blot of TLR4 pathway proteins. The expression of TLR4, phospho–NF‐κB, NF‐κB and ICAM1 was detected by Western blot analysis. D, MMP of the mitochondria extracted from lung tissue was examined by FCM. E, Representative HE staining images of the lung in the four groups (original magnification, ×400). F, Evans blue staining. G, IL‐6 level in BALF. H: Lung W/D ratio. *P < .05 vs the C group, *#P* < .05 vs the MV group. Experiments were repeated at least three times

NLRP3 and p120 in the lung tissues were determined by Western blot analysis, and inflammatory cytokines were determined by ELISA. The results revealed that the NLRP3 expression was increased in the MV group compared with the C group, whereas this effect was reversed in the MCC950 pre‐treatment group (Figure 5A) (*P* < .05). HE staining indicated that normal alveolar wall structure was destroyed in the MV group, whereas this effect was reversed in the MCC950 pre‐treatment group (Figure 5B). The expression of p120 was decreased in the MV group compared with the C group, and this effect was enhanced after 4 hours of high tidal volume ventilation in the p120 KO group. However, the expression of NLRP3 was increased in the MV group and p120 siRNA group compared with the C group, while NLRP3 expression was most significantly enhanced in the p120 siRNA + CS group (Figure 6A b,c) (*P* < .05). Western blot analysis revealed that the expression levels of TLR4, phospho–NF‐κB, NF‐κB and ICAM1 were all increased in the p120 siRNA group and CS group, while they were enhanced in the p120 siRNA group after 4 hours of 20% cyclic stretching (Figure 6C).

As shown in Figure 6D, mechanical ventilation can reduce MMP, and MMP was also decreased in the group treated with p120 siRNA compared with the C group. Moreover, the decrease in MMP was enhanced after high tidal volume ventilation. HE staining indicated that the alveolar structure was destroyed, as demonstrated by thickened and ruptured alveolar wall and disorderly arrangement of the nuclei of the alveolar epithelium in the MV group and p120 KO group. These effects were aggravated in the p120 KO group under high tidal volume ventilation (Figure 6E). Additionally, Evans blue staining revealed deep colour dyeing in the MV group and p120 KO group, which suggested permeability in these two groups. This permeability was further increased in the p120 KO + MV group (Figure 6F). Similarly, the W/D ratio, which reflected pulmonary oedema, was increased in the MV, p120 siRNA and p120 siRNA + MV groups compared with the C group. (Figure 6H) (*P* < .05). Moreover, the production of IL‐6 and IL‐1β in BALF was significantly increased in the MV, p120 KO and p120 KO + MV groups compared with the C group, which indicated pulmonary inflammation (Figure 6B and G) (*P* < .05).

## 4 DISCUSSION

**Figure 7:**
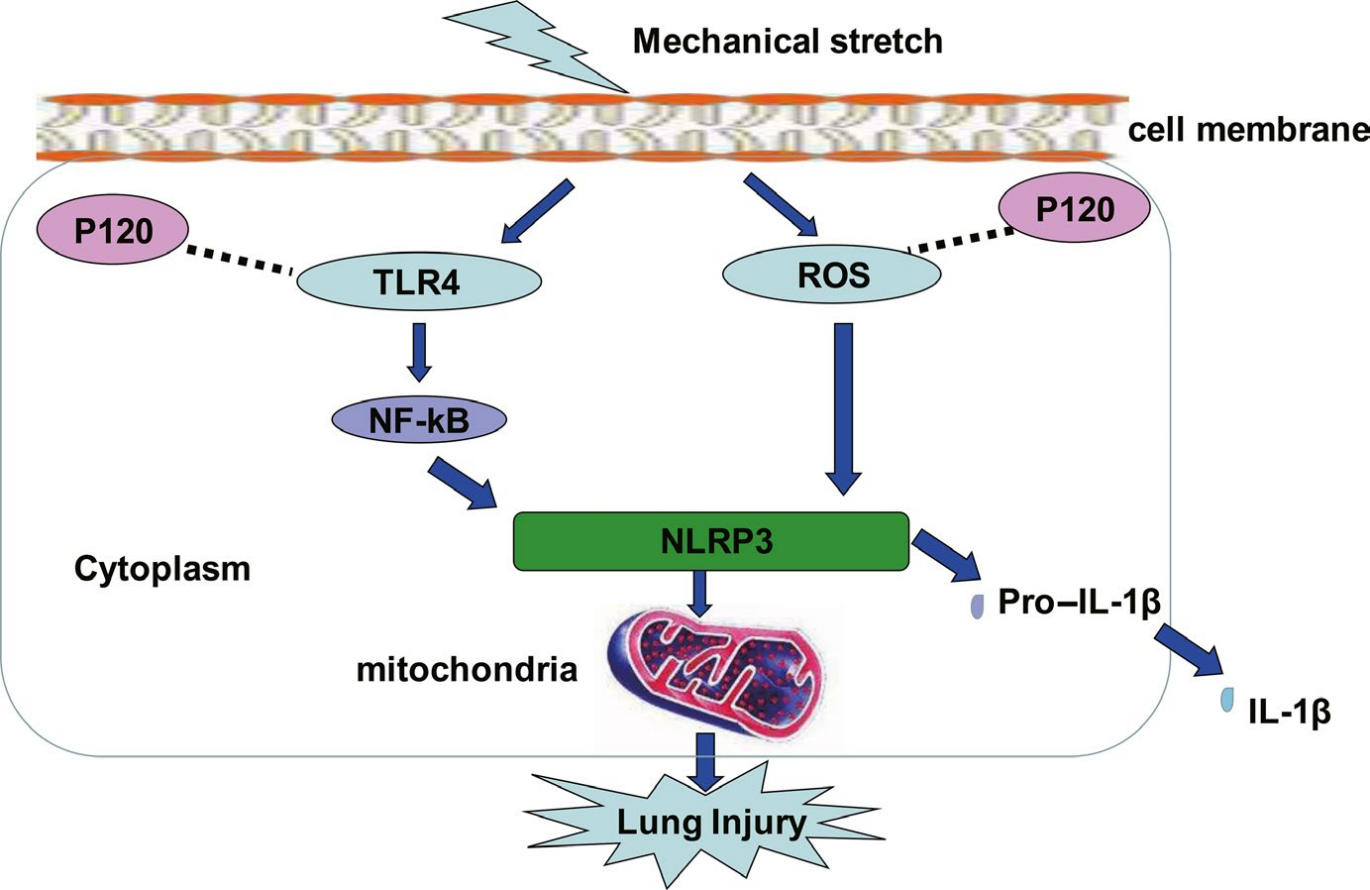
Mechanisms of p120‐mediated regulation of epithelial ventilator‐induced lung injury

VILI is characterized by pulmonary oedema caused by the destruction of cell barrier integrity. Mitochondria, as the main site of energy production, provide energy to support for the maintenance of alveolar membrane integrity. NLRP3 is involved in the process of VILI. As an important cell junction protein, p120 plays a role in strengthening the firmness of intracellular cytoskeletal protein, thus preventing the increase in pulmonary vascular permeability and reducing the incidence of pulmonary oedema Figure 7.

In this study, we exposed MLE‐12 cells to cyclic stretching (Flexcell 5000T) to establish the VILI model, which was successfully verified by a previous study.^6^ The results revealed that cyclic stretching could destroy the structure and function of mitochondria (Figure 1A) and increase the level of NLRP3 (Figure 1B). MMP was increased when we pre‐treated MLE‐12 cells with the NLRP3 inhibitor MCC950 compared with cyclic stretching alone (Figure 1A). Therefore, we concluded that NLRP3 may play an important role in regulating mitochondria in VILI. A recent study showed that inhibiting NLRP3 inflammasome activation ameliorated VILI.^24^ In that study, the researcher explored the role of three components of NLRP3 inflammasome (NLRP3, caspase‐1 and ASC) in VILI and p120 was simply used as an indicator that VILI could disrupt cell junctions. However, our study is very different from that researchers'. What we focus on is that p120, as a key upstream factor, acts on NLRP3 protein of NLRP3 inflammasome and plays a protective role on mitochondria in VILI. Besides, our study has suggested that ROS production in response to cyclic stretching was related to the up‐regulated expression of NLRP3. Mechanical stretching activates NLRP3 inflammasome via a mitochondrial ROS‐dependent signalling pathway.^14^ Two studies have shown interactions between NLRP3 and ROS.

In addition, mitochondria were also destroyed after p120 depletion with or without cyclic stretching (Figure 4A,B). Mitochondria are the main source of energy for the whole cells. Mechanical force, inflammation and other harmful stimuli can all affect mitochondria.^25^ Mitochondrial respiratory dysfunction, changes in enzymatic activity, mitochondrial ROS generation and structural damage can be involved in this process.^26^, ^27^, ^28^ The results of this study were similar to those of previous studies.

NLRP3 markedly increased after p120 depletion with or without cyclic stretching (Figure 2C). However, the possible interaction between p120 and NLRP3 remained unknown. Recent studies have reported that p120 inhibits the TLR4 and MyD88 signalling pathways to inhibit inflammation.^16^ p120 also plays a protective role in oxidative stress.^29^, ^30^ During the inflammatory response induced by VILI, the production and regulation of NLRP3 are related to the phosphorylation of NF‐κB induced by TLR4. Phosphorylated NF‐κB can promote the synthesis of NLRP3 inflammasomes by up‐regulating the expression of NLRP3, which increases the release of IL‐1β and aggravates inflammation.^31^, ^32^, ^33^, ^34^ Therefore, we determined the possible mechanism of p120 in regulating NLRP3 in this study. We detected the activity of NF‐κB, phospho‐NF‐κB and ICAM1, which represent the TLR4 signalling pathway. The results suggested that the TLR4 pathway was activated after p120 depletion with or without cyclic stretching, which was similar to the changes in NLRP3 (Figure 2D and Figure 3A,B and 3). Although this mechanism was not fully demonstrated, some important relationships between p120 and NLRP3 have been clarified.

Moreover, these mechanisms were verified in vivo and with other indicators (IL‐1β, IL‐6, W/D ratio, Evans dye, HE staining) reflecting lung injury after mechanical ventilation. The results of W/D ratio, Evans dye and HE staining all indicated different degrees of pulmonary oedema after pre‐treatment with p120 siRNA‐liposomes with or without mechanical ventilation. Above all, we concluded that p120 may protect mitochondria by inhibiting NLRP3 activation in VILI. This experimental study also needs to be improved. In animal experiments to verify this experiment, the use of isolated and purified mouse lung epithelial cells will improve the accuracy and rigour of the research. However, lung tissue was chosen because of the technical difficulty of isolating mouse lung epithelial cells.

This study demonstrated that mechanical ventilation could destroy the structure and function of mitochondria by activating NLRP3. In addition, p120 could protect mitochondria by inhibiting TLR4 pathway‐induced NLRP3 activation in VILI. Based on the above results, we suspected that p120 might play a key role in initiating a series of downstream reactions as an essential messenger in VILI. Considering the mitochondrial energy productivity of the cells, potential therapeutic approaches to protect mitochondria represent a new idea for the treatment of VILI.

## CONFLICT OF INTEREST

The authors declare that they have no competing interests.

## AUTHORS' CONTRIBUTIONS

Yuelan Wang designed the research; Ge Liu conducted the experiments, drafted the manuscript and prepared the figures; Changping Gu, Huan Liu, Dong Wang, Xiaobin Liu and Yuelan Wang edited and revised the manuscript; Changping Gu and Mengjie Liu analysed the data; Yuelan Wang interpreted the results of the experiments and approved the final version of the manuscript. All authors read and approved the final manuscript.

## Data Availability

The data sets used and analysed during the current study are available from the corresponding author on reasonable request.
